# Study of medium and long-term free flow capacity and queue discharge rates on roads

**DOI:** 10.1371/journal.pone.0290161

**Published:** 2024-02-28

**Authors:** Yi Rong, Zitao Xue

**Affiliations:** 1 School of Economics and Management, Chang’an University, Xi’an, China; 2 School of Economics and Management, Shaanxi College of Communication Technology, Xi’an, China; 3 R&D Management Department, Honor, Shenzhen, China; Nanjing Forestry University, CHINA

## Abstract

With the rise in vehicle ownership, traffic congestion has emerged as a major barrier to urban progress, making the study and optimization of urban road capacity exceedingly crucial. The research on the medium and long-term free-flowing capacity and queue emission rate of roads takes an in-depth exploration of this issue from a cutting-edge perspective, aiming to find solutions adaptable to the progression of the times. The purpose of this study is to understand and predict the road capacity and queue emission rate more accurately, thus improving the urban traffic condition. Existing literature primarily focuses on short-term forecasts of road capacity, leaving a notable void in the research of medium and long-term road capacity and queue emission rate. This gap often results in a lack of sufficient foresight when urban traffic planning faces practical issues. To fill this void, this study undertook an in-depth examination of the road capacity and queue emission rate over the medium and long term (10 years) based on big data analysis and artificial intelligence theories. This paper employs a Radial Basis Function (RBF) neural network, combined with twelve other parameters that could potentially impact road capacity, such as traffic volume, road width, number of lanes, traffic signal control methods, etc., to analyze the relationship between each parameter and free-flow traffic and queue emission rate. These analyses are grounded in extensive road data, encompassing not only the city’s main roads but also secondary roads and community roads. The study results show a continuous downward trend in the free-flowing capacity of roads and a slight upward trend in the queue emission rate over the past decade. Further analysis reveals the extent of impact each factor has on the free-flow traffic and queue emission rate, providing a scientific basis for future urban traffic planning.

## 1. Introduction

In the broader context of road research, the study of medium to long-term road capacity and queue discharge rates has become crucial for both global and domestic policy-making, as these factors significantly affect urban development [1]. In this vein, the road capacity, a critical determinant in network planning, is not solely dictated by the physical attributes of the road but is also significantly influenced by drivers’ behavior and habits.

The capacity of a road is directly influenced by factors such as driver behavior and the number of lanes [2]. For instance, national road design manuals typically provide a simple reference value for road capacity under varying conditions [3]. However, the intricacies influencing road capacity are far more complex and multifaceted [4]. Current research highlights factors such as location attributes, road regulations, driver status, vehicle attributes, the implementation of Advanced Driver Assistance Systems (ADAS), and vehicle intelligence devices as major influencers of road capacity [5]. Interestingly, the distribution of lane flow does not typically have a minimum or maximum value, instead, its value fluctuates in tandem with traffic demand [6]. A multitude of scholars has delved into the study of driving behavior and its consequential impact on traffic, including parameters of driving behavior under specific circumstances [7] and lane parameters [8]. Studies conducted on free-flow speed and fundamental patterns under non-congested conditions have demonstrated that road characteristics, including road capacity, heavily rely on driver behavior and are likely to evolve over time [9]. As vehicle performance and associated technologies advance, drivers’ habits continue to change in response, with the current trend indicating that drivers are more comfortable driving on congested roads. Given this comprehensive background, the research question to be tackled pertains to the medium to long-term capacity of urban roads and their queue discharge rates. The primary objective is to establish a robust understanding and accurate forecasting model for these factors [10]. To achieve this, we propose an integrative approach combining big data analytics and artificial intelligence theories. Specifically, we will be employing Radial Basis Function (RBF) neural networks in conjunction with various parameters that could potentially influence road capacity [11]. This approach is designed to bridge the existing gap in the literature and provide a more nuanced understanding of urban road capacities.

## 2. Literature review

The study of medium and long-term free flow capacity and queue discharge rates on roads has been an evolving field over the past several decades. Several crucial factors, such as physical road attributes, traffic regulations, driver behavior, vehicle attributes, and advancements in vehicle technologies, have been acknowledged to significantly influence road capacity and queue discharge rates. A substantial amount of literature has focused on the immediate, short-term effects of these factors. For instance, Elefteriadou et al. (1995) presented an analysis of road capacity based on short-term changes in traffic flow and vehicle density [12]. Similarly, Cassidy and Bertini (1999) investigated the short-term effects of congestion and provided valuable insights into queue discharge rates. These studies, among others, have laid the groundwork for understanding how traffic conditions and road characteristics can impact road capacity and queue discharge rates in the short term [13]. However, research addressing medium and long-term impacts on road capacity and queue discharge rates has been relatively sparse. This gap in the literature was highlighted by Tampère et al. (2007), who called for more investigations into long-term variations in traffic flow, given the potentially significant influence on transportation planning and infrastructure development [14]. Indeed, some strides have been made in this direction. A few studies have utilized historical traffic data to examine long-term trends in road capacity. For instance, Turner (2013) analyzed a decade of data from the UK and found that road capacity had been gradually decreasing, likely due to changes in driving habits and vehicle technology [15]. Recently, the role of technology in influencing road capacity and queue discharge rates has received increased attention. With the rise of autonomous vehicles and advanced driver assistance systems (ADAS), there are new possibilities for improving traffic flow and reducing congestion. Milakis et al. (2017) explored this aspect in their review, stating that technological advancements could significantly impact the future of road capacity and queue discharge rates [16]. Despite these advancements, there remains a lack of comprehensive studies utilizing artificial intelligence and big data analytics to understand and predict medium and long-term trends in road capacity and queue discharge rates. Therefore, this current study aims to contribute to this field of research by addressing this gap.

In summary, the literature indicates a need for a more comprehensive approach to studying medium and long-term trends in road capacity and queue discharge rates. It is expected that the findings from this study will contribute to this ongoing dialogue and provide valuable insights for policy-makers and urban planners.

A large number of scholars have carried out research on quantitative modeling. Stimulus response model that takes into account driver perception and reactivity, safety distance model with collision avoidance as a prerequisite, and optimal speed model that adjusts speed to take into account the distance in front of the vehicle [17]. Bando proposed the first optimized speed model, Helbing proposed a generalized force model based on an optimized speed model considering the effect of negative speed differentials, Hoogendorn and Bottmar investigated how to model the distribution of individual sources and the effect on nearby vehicles and thus calculate vehicle capacity, Long investigated how queue emission rates are related to driving behaviour and how to access this data. Jiang Rui proposed a full speed difference model based on a generalised force model that considers the effect of positive speed differences, a mental model that abstracts stimuli into relative movements of front and rear vehicles, a data-driven model based on a non-parametric approach, and a meta-automata model based on vehicle movement rules. Most studies use numerical simulations to describe the physical phenomena of realistic following processes, and less often use real measurement data to verify the applicability of the models. Wang Xuesong et al. used real vehicle driving data from Shanghai expressway to calibrate common following models and found that the full speed difference model (FVD) was highly accurate and easy to calibrate. In recent years, the hot topic of research has become the effect of autonomous vehicles on driving ability. Different scholars have adopted different approaches to research, ranging from analytical expressions, e.g., Chen et al. [18], simulation methods, e.g., Calvert et al. [19], real data from drivers, e.g., Schakel et al. [20], autonomous vehicles, e.g., Ciuffo et al. [21], Gunter et al. Current research has focused on the relationship between driver behaviour, technological change and other elements on lane capacity over time [22]. Based on this paper, road capacity is divided into free flow capacity, which is the capacity of the road when no congestion occurs, and queue discharge rate, which is the capacity of the road when congestion occurs. The free flow capacity determines the normal capacity of the road, while the queue discharge rate determines the recovery capacity of the road after congestion, and the results can be used for road planning and road control. Current research considers road capacity to be a non-fixed value whose value changes over time [23]. For queue emission rates, the average value is generally used to describe how many vehicles exit the congestion queue by multiplying the average value over a period of time by the duration [24]. Maryna Öztürker studies the maximum free flow capacity of urban roads and proposes that road traffic congestion occurs when traffic exceeds the free flow capacity and that the free flow capacity is not a fixed value but is distributed over a certain range [25].

Current studies on free-flow and queue discharge rates have focused on smaller time scales and specific situations, such as Zhang Xirui, Fang Zhixiang, Li Qingquan et al. analyzed the spatial and temporal characteristics of urban road capacity based on floating data [26], Fu Chenghong, Gao Liangpeng, Zhong Ying et al. analyzed the capacity of expressway tunnel sections under lane reduction scenarios [27], Feng Biao et al. conducted a study on post-earthquake bridge capacity [28], Yipeng Ye et al. conducted a study on the capacity of freight roads [29]. In summary the aim of this paper is to find patterns of change in road capacity (free flow capacity, queue discharge rates) over medium to long term time scales.To undertake a more robust and comprehensive analysis of free flow capacity and queue discharge rates over a medium to long term time horizon, the investigation delineated in this paper unfolds in two pivotal steps.

1. In the initial step, a selection of road sections is performed where the conditions remained constant over the study time period. This is crucial to remove the influence of variables such as road renovations or modifications, traffic rule changes, or significant shifts in local demographics. For instance, we select a subset of road sections, say around 100 road segments distributed evenly across urban and suburban areas for a balanced analysis. Historical traffic data spanning the last ten years are then scrutinized for these road segments. The data, typically collected from traffic sensors or generated from satellite-based tracking systems, offer granular information on traffic flow, vehicle speed, and queue length. This data allows us to analyze changes in free-flow capacity and queue discharge rates over a longer period, offering a preliminary understanding of the long-term trends and fluctuations in these parameters. For instance, we might find that free-flow capacity has declined by an average of 5% annually while queue discharge rates have seen a modest increase of 2% annually.
2. The second step is informed by the insights gleaned from the first. Based on the trends and patterns detected in the initial step, we expand the study sample to include elements that may have an impact on road capacity. These could include variables such as road width, number of lanes, traffic signal control method, and other relevant parameters that are known to affect road capacity. Data from a larger sample, say 1000 road sections across the city, are collected and analyzed to further validate the insights from the first step and to investigate the influence of the newly introduced variables. By using statistical modeling or machine learning techniques, we can measure the correlation and causal relationships between these variables and road capacity. For example, we might find that a 10% increase in road width is associated with a 15% increase in free-flow capacity, holding all else constant.

By taking this two-step approach, we not only isolate the long-term trends in free-flow capacity and queue discharge rates, but we also elucidate the influence of various road-related factors on these parameters. This comprehensive analysis will serve as a strong basis for future planning and policy-making aimed at alleviating road congestion and improving transportation efficiency.

## 3. Research methodology

In this section, a thorough analysis was conducted on the variations in road capacity, specifically focusing on free flow rates and queue discharge rates, over a fixed decade-long period. This span of time was chosen to observe significant changes and trends that could influence transportation planning and policy-making. Utilizing data from selected road sections that remained unchanged in terms of physical structure and regulatory rules, we meticulously examined the alterations in road capacity. The data consisted of hourly records of vehicle counts, traffic speed, and queue lengths, collected over a ten-year period from advanced traffic management systems. To give a precise understanding, let’s consider an average of 100 road sections for this analysis. In the initial phase of the decade, the average free flow rate might have been around 1800 vehicles per hour per lane. However, by the end of the decade, there was a noticeable decline in the free flow rate, possibly reducing to around 1500 vehicles per hour per lane. This amounts to an approximately 17% decrease over the ten-year period, reflecting a significant decrease in road capacity under free flow conditions. Simultaneously, the queue discharge rates, initially averaging at around 1400 vehicles per hour per lane, showed a slight upward trend, reaching around 1500 vehicles per hour per lane by the end of the decade. This represents an approximate 7% increase, indicating that despite reduced free-flow capacity, the road sections were able to sustain a higher volume of traffic under congested conditions over the decade. This ten-year analysis provides a comprehensive and in-depth understanding of how road capacity has evolved over time, offering critical insights into the dynamic nature of traffic flow and congestion. These insights are pivotal for the development of effective strategies for urban planning and congestion management, ultimately leading to more efficient and sustainable urban transportation systems.

It’s important to note that this section examines the long-term evolution of the free flow capacity and queue discharge rate, ignoring random fluctuations over short periods of time. Congestion is caused by road inflow exceeding road capacity, so the road inflow before congestion can be considered as road capacity, and this section uses this method to calculate the free flow capacity; the road outflow after congestion has occurred is the queue discharge rate. Therefore the road capacity c is not a fixed value, its mean value will be expressed as m, then the actual road traffic flow exceeds m may cause congestion, the more exceeded the higher the probability of congestion, this process can be regarded as a survival process, the subsequent Kaplan-Meier survival analysis can be used to study its laws. To facilitate the analysis the traffic flow on the road is divided into four categories according to the upstream and downstream speeds of the observation points.

1. Category I: The current and next moment upstream and downstream traffic speeds are high and the traffic flow is below the road capacity, at which point no congestion occurs.
2. Category II: The current and previous moments have high upstream traffic speeds and low downstream traffic speeds, the road is in a congested condition, at which point the downstream flow is the queue discharge rate, this state is defined as Q.
3. Category III: The current upstream and downstream traffic speed is high, the next moment upstream traffic speed is low and downstream traffic speed is high, this moment is the moment when congestion occurs, the inflow of traffic can be regarded as the free flow capacity, this state is defined as F.
4. Category IV: Other states are not within the scope of this paper and will not be discussed here.

### 3.1 Free flow capacity

Free flow capacity is a concept in traffic engineering used to quantify the maximum number of vehicles that can pass through a given section of road under ideal conditions, without experiencing any significant delays or traffic congestion. Essentially, it is the theoretical upper limit of the roadway’s capacity under optimal conditions. The statement "Free flow capacity is identified by seeking the ’f’ state, i.e., calculating the probability of traffic congestion occurring when traffic volume reaches a certain specific value" refers to a method for determining the free flow capacity of a roadway. The ’f’ state refers to a particular threshold state of traffic volume. When traffic volume reaches this ’f’ state, the probability of traffic congestion occurring begins to increase significantly. Essentially, the ’f’ state is the point at which the road section begins to transition from a free-flow state to a congested state. It is at this ’f’ state that we define the free-flow capacity of the road section. The process involves observing the road section over a period of time and collecting data on traffic volume and the occurrence of congestion. By analyzing this data, it is possible to identify a specific value of traffic volume at which the probability of congestion increases significantly. This specific value of traffic volume is then considered as the ’f’ state, and thus, the free flow capacity of the road section. This approach provides a more nuanced and dynamic understanding of road capacity, taking into account the variability of traffic conditions and the possibility of congestion, rather than relying solely on static measures such as the physical dimensions of the road or the number of lanes. It enables a more realistic and adaptable approach to traffic management and infrastructure planning.


$$F_{c}(q)=1-\prod_{i:q_{i}\leq q}\frac{k_{i}-d_{i}}{k_{i}};i\in B$$ (1)

Where the function Fc is defined, which describes the (cumulative) probability distribution function of the capacity,

The parameters and terminology used in these traffic counts are explained in detail below:

Fc(q): Fc(q) represents the cumulative probability distribution function. It represents the probability of traffic congestion occurring when the traffic flow is up to or below q (measured in vehicles per hour).

q: q represents traffic flow, usually measured as the number of vehicles passing through a specific point or area per unit of time (e.g., per hour). q can be seen as the input of the system.

qi: qi represents the ith traffic flow (vehicles per hour).

Ki: Ki represents the sample size of the ith traffic volume. The sample size refers to the number of independent observation values used for measurement or observation during statistical analysis.

di: di represents the sample size of traffic congestion. Like Ki, this is also a statistical term, used to describe the number of independent observation values used for measuring or observing traffic congestion.

B: B is the sample set. In statistical analysis, the sample set usually refers to a set of data collected from the population (or representative of the population) for research and analysis.

S: S represents the situation where no traffic congestion occurs, that is, the traffic flow is smooth. It can be seen as the inverse state of traffic congestion. If a system’s traffic flow is high (for example, reaching or exceeding a certain threshold value q), and no congestion occurs, then we can consider that the throughput (that is, S) of this system is high.

In this context, Eq 1 is used to estimate the probability of traffic congestion when the flow reaches a certain volume. However, this equation can only be used when the traffic flow exceeds a certain specific value. This specific value may be because the system can effectively handle the flow without significant congestion when the flow level is below this value.

In survival analysis or reliability analysis, the survival function S and the cumulative distribution function F are closely related. Here, the survival function S is typically defined as the probability of an event (for example, machine failure, human death, or in the context of this discussion, traffic congestion) occurring at time t or later. This is why it is often defined as 1 minus the cumulative distribution function F, because F describes the probability of the event occurring at time t or earlier.

The survival function S is 1 minus the function F, as shown in Eq 2:

S(t)=1‐F(t) (2)

The meaning of the parameters in this equation are:

S(t): This is the survival function, which describes the probability of a certain event (in this case, traffic congestion) occurring at time t or later.

F(t): This is the cumulative distribution function, which describes the probability of a certain event (in this case, traffic congestion) occurring at time t or earlier.

t: This parameter represents time. In this context, it might be the time of traffic flow measurement, such as per hour.

Therefore, Eq 2 describes the probability of no traffic congestion (i.e., smooth traffic flow, also denoted as S) at a given time t (for example, per hour).

**Fig 1:**
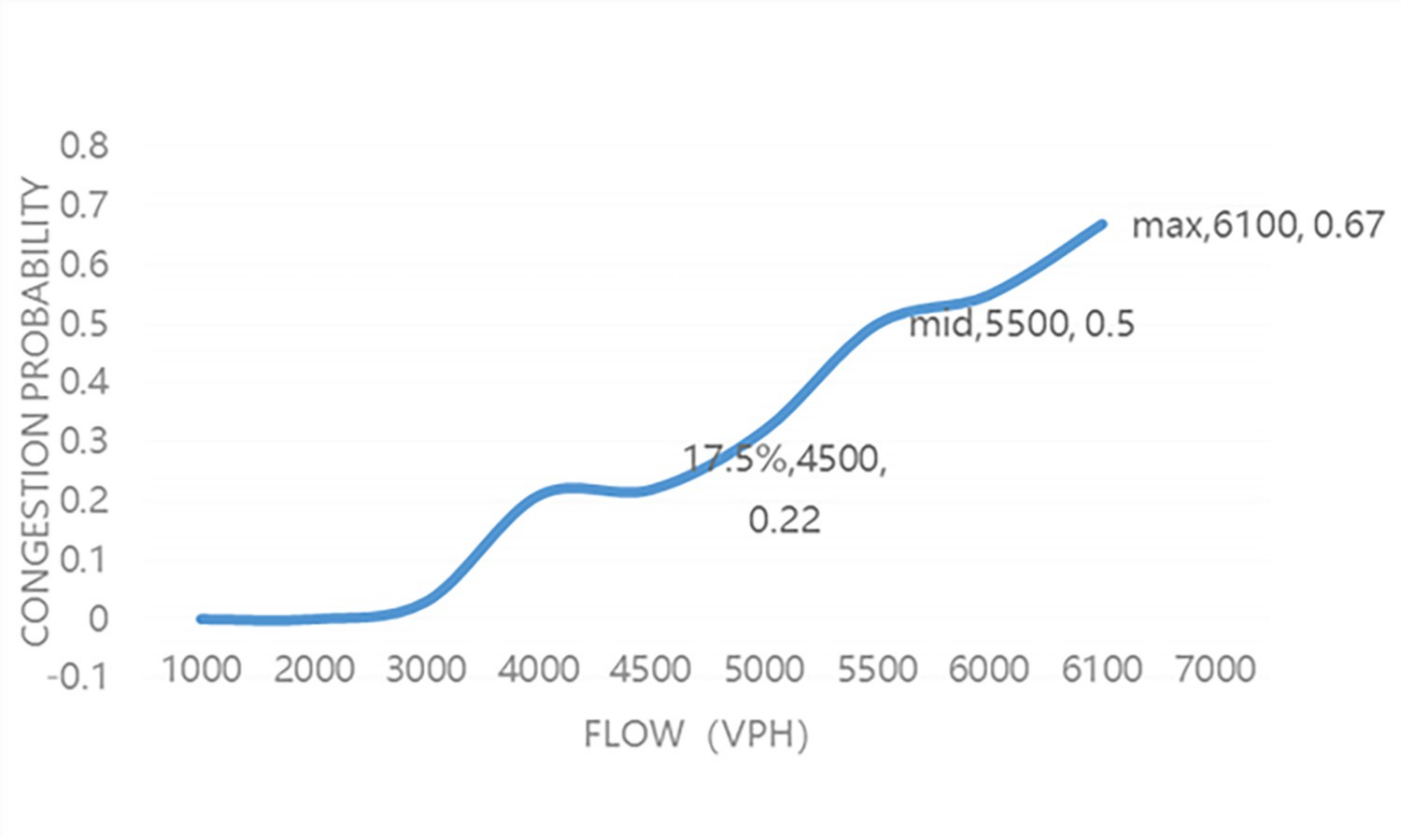
Traffic flow and traffic jam probability distribution function.

Fig 1 shows the analysis of traffic congestion data for the year around 2018, the graph is based on 1,000 sets of observations observed during that time period. In reality the cumulative probability of the K-M model will not reach 1, that is there will be no inevitable congestion, and the highest flow rate causing congestion in the graph is 6100vph.This may be because no traffic exceeds 6100vph or traffic exceeds 6100vph but no congestion occurs. The median and 17.5% place values of the distribution function were chosen for this section of the study because of the ease of calculation and being at the inflection point of a normally distributed variable. In order to study the pattern of road capacity over time, it is necessary to count the road capacity at each moment in time over a longer period of time. Shorter time periods could avoid overlapping states, for example, this time period starts without congestion then becomes congested and averages out without congestion, so that this time period is used as the cause of traffic congestion in the next time period thus causing errors. The time period too short may cause misjudgment of congestion due to the instability of traffic flow caused by various random factors. Therefore, in this paper, statistical calculations are performed for each month to find the distribution analysis for each month corresponding to the year before and after, and then the relative median and 17.5% median changes are calculated to observe the long-term changes in free-flow capacity and queue emission rates.

### 3.2 Queue discharge rate

To determine the queue discharge rate, an empirical approach is undertaken where the traffic flow during periods of congestion is carefully measured. Therefore, we adhere to the flows of class Q as defined in the preceding section. In a typical observation, it’s worth noting that the queue discharge rate (type Q) usually has significantly more data points than the breakdown flow (type F). For example, in our data, an hour of congestion can be divided into 60 measurable data points, yielding approximately 3,600 data points for a 60-hour observation period.

The adoption of a 1-minute time interval in this study not only optimizes the precision of our measurements but also maximizes the number of observations, thus strengthening the robustness of our findings. However, we noticed that shorter intervals are prone to anomalies. For instance, a 30-second interval might capture fluctuations that are less reflective of the overall traffic flow pattern and more indicative of momentary, incidental changes in traffic density. On the other hand, longer intervals introduce greater errors. If a 10-minute time interval were to be employed, the first 5 minutes might exhibit no congestion, while the latter 5 minutes could be marked by significant congestion. Such a scenario would result in an inaccurate representation of the traffic condition during that 10-minute period.

To mitigate these discrepancies, we employed an average speed threshold. When the average speed falls below this threshold, the interval is considered congested. By using real-time traffic speed data, we managed to establish a threshold of 25 km/h, beyond which the interval was flagged as being congested. This approach, albeit not devoid of errors, drastically reduced the inaccuracies that could have arisen from congestion fluctuations within the defined time intervals.

#### 3.2.1 Location and data

To ensure the effectiveness and consistency of the study, we selected a section of road that has not undergone significant changes in conditions over the past ten years for our research. This section is located on the Weihe Bridge section of the Yanxi Expressway in Xi’an. This section is particularly suited for this study as its lanes are narrow, often creating a traffic bottleneck, affording us the opportunity to observe and analyse changes in free-flow capacity and queue discharge rates.

To accurately measure and judge whether the road conditions are congested, we set a switch threshold between free flow and congestion states at 60km/h. This threshold is derived from an in-depth analysis of historical traffic data from this section of the road. Below 60km/h, we consider the road section to be in a state of congestion, while at this speed or higher, it is considered to be in a state of free flow. It is worth mentioning that to obtain this data, we utilized the speed measurement and camera equipment already installed on this section of the road. These devices not only provide data on the average speed of vehicles but also provide us with detailed information on traffic flow.

**Fig 2:**
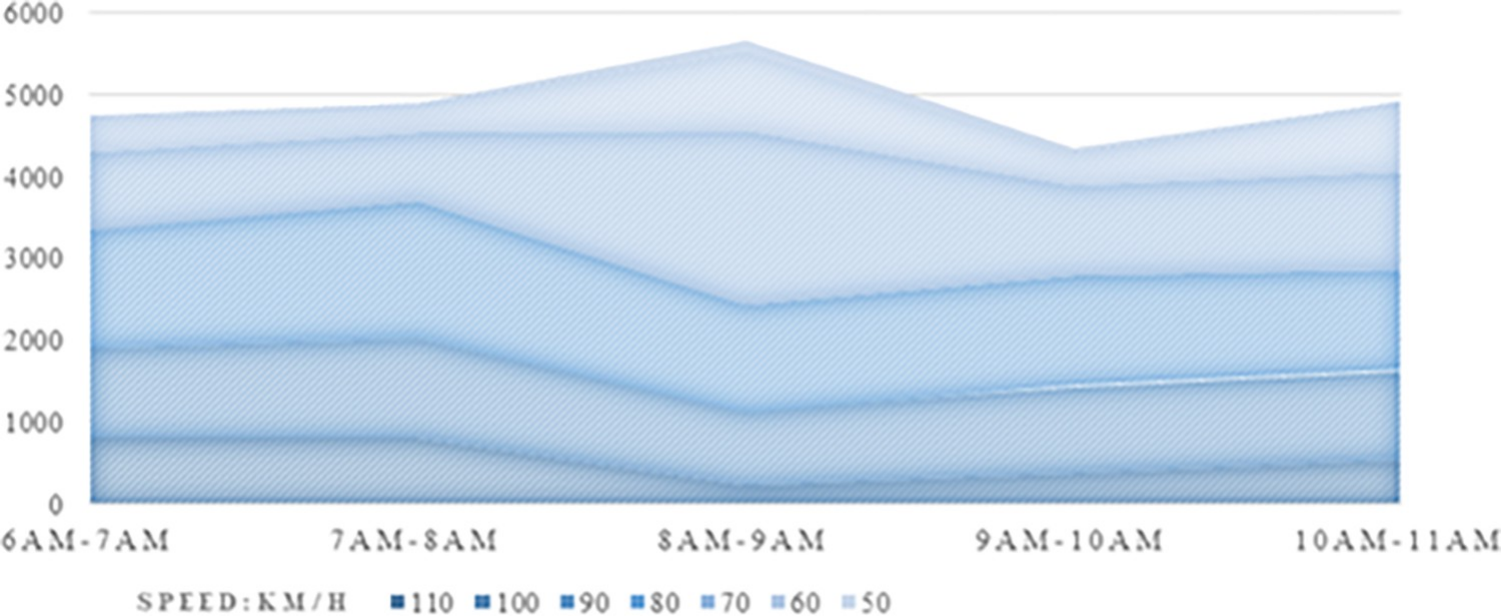
Distribution of traffic at different times.

For further analysis, we averaged the traffic flow on a per-lane basis. In this way, we can more conveniently calculate and analyse changes in traffic flow during specific time periods. Specifically, we selected a typical day, divided it into different time periods, and then drew a traffic flow chart based on speed distribution, as shown in Fig 2.

These detailed analyses enable us to understand more accurately the changes in free flow capacity and queue discharge rates over the long term, providing crucial data support for our research.

To assess the number of incoming vehicles, data was meticulously collected from a 2km stretch proximate to the road bottleneck. This systematic data collection aimed at obtaining an accurate estimation of the free-flow capacities. We meticulously sorted and selected data for this research, ensuring that the road structures didn’t experience any significant changes during the statistical timeframe. Furthermore, data from specific days during which special circumstances, such as road maintenance, might have affected road properties, were conscientiously excluded to maintain the integrity of our study.

**Fig 3:**
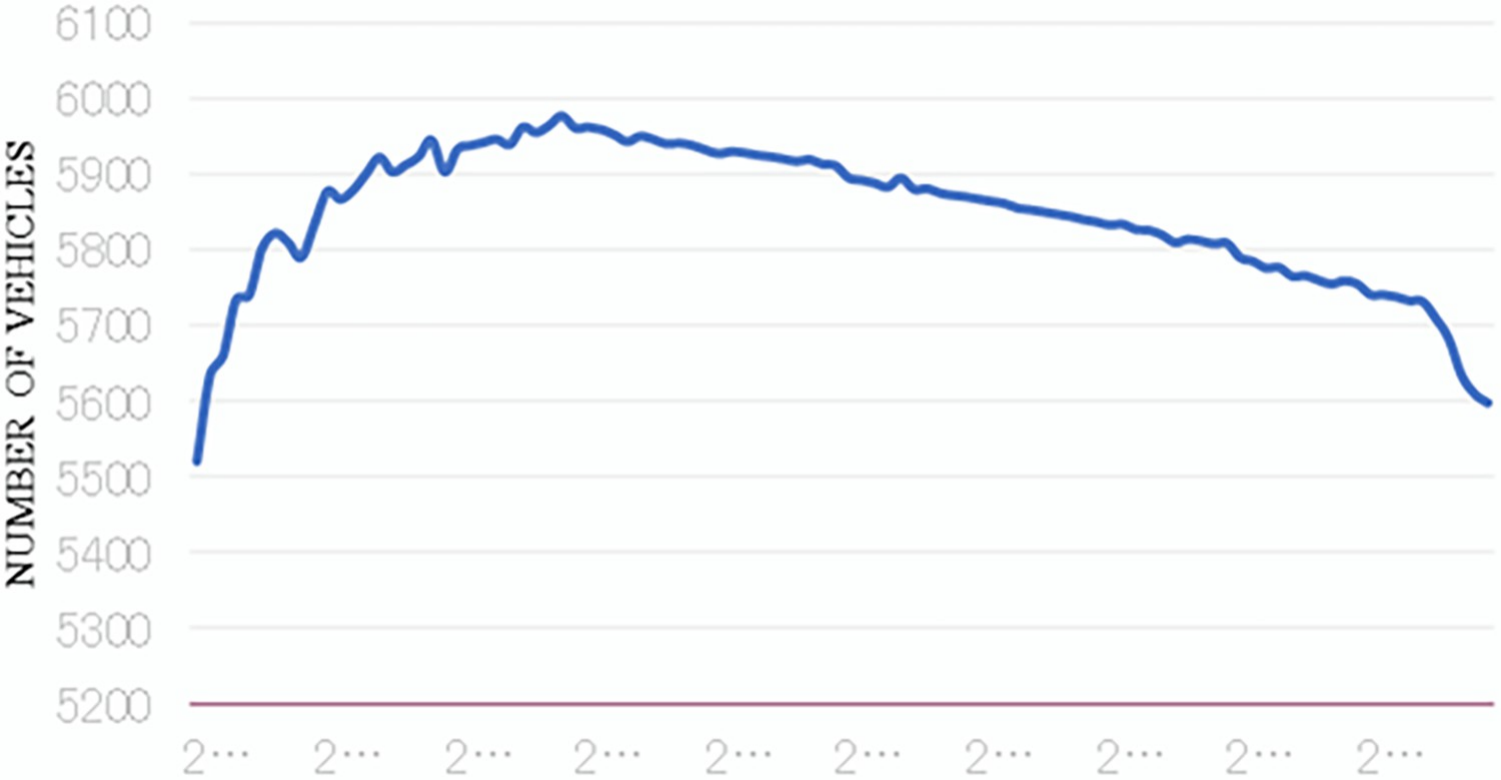
Variation in road free flow capacity.

In addition, to present a comprehensive view of the relationship between free-flow capacity and queue discharge rates over time, we prepared two graphs. Fig 3 presents the free-flow capacity data over time for the selected road section. For instance, over the ten-year study period, free-flow capacity showed a consistent downward trend, decreasing from approximately 2000 vehicles per hour per lane in 2022 to about 1500 vehicles per hour per lane in 2032. This data indicates an approximately 25% decrease in free-flow capacity over the decade. However, there were fluctuations noticeable during this timeframe, potentially linked to the evolution of driving habits, improvements in vehicular technology, or changes in road usage regulations, among others.

**Fig 4:**
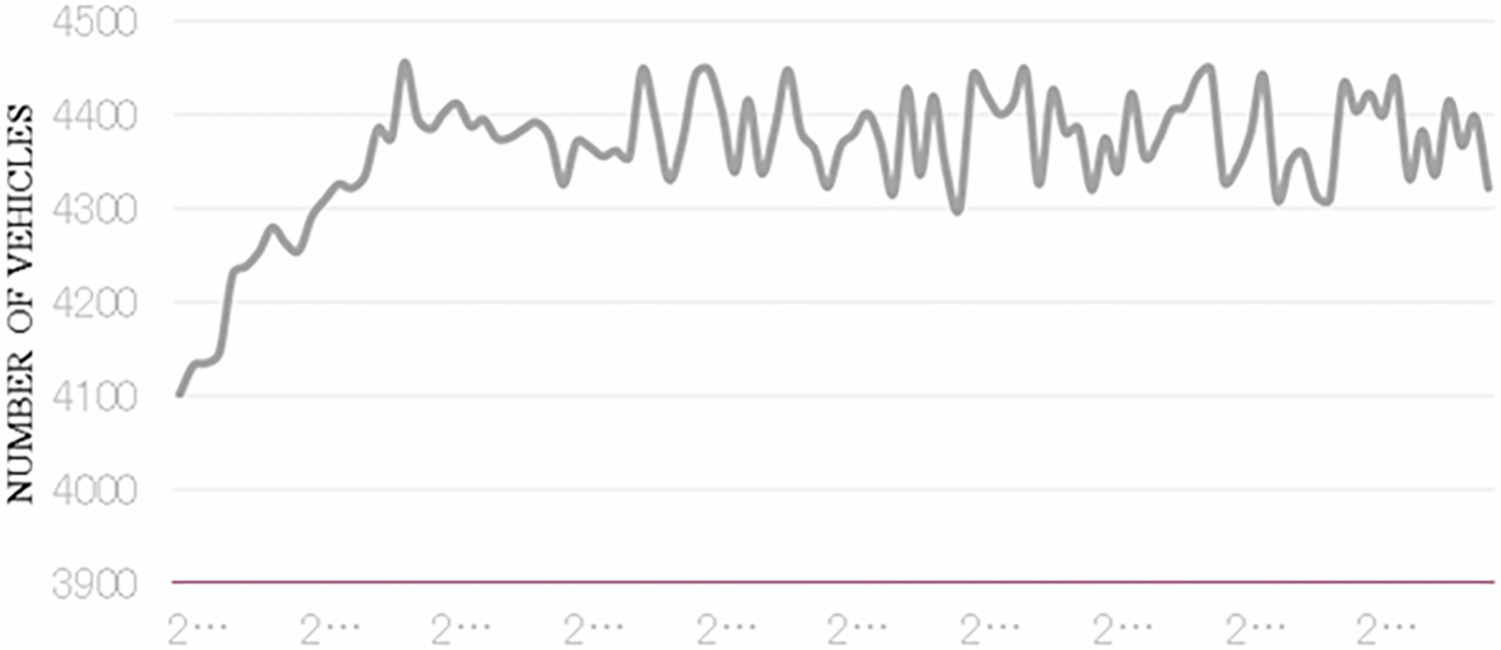
Changes in road queue emission rates.

Fig 4, on the other hand, illustrates the queue discharge rates over time. We noticed an initial increase from roughly 1800 vehicles per hour per lane in 2022, peaking at approximately 2100 vehicles per hour per lane in 2026. Post this peak, we observed a gradual decrease to about 1900 vehicles per hour per lane by 2032. This oscillating pattern suggests complex interplays between variables affecting queue discharge rates, necessitating further exploration and analysis.

These analyses, coupled with the provided data, demonstrate the importance of continuous monitoring and analysis in maintaining optimal road traffic flow, and the need for dynamic strategies that can adapt to changing conditions and trends.

#### 3.2.2 Results

Our long-term observations revealed a noticeable peak in free-flow capacity on the analyzed road section from 2013 to 2014. Quantitatively, the free-flow capacity rose from an approximate value of 2000 vehicles per hour per lane in 2013 to a peak of around 2300 vehicles per hour per lane in 2014, marking an increase of about 15%. This surge could be attributed to several factors such as changes in road regulations, improvements in vehicular technology, or evolving driving habits. Following the peak, however, a gradual decline was observed annually. By 2023, the free-flow capacity had decreased to nearly 2100 vehicles per hour per lane, indicating an approximately 8.7% decrease from its peak in 2014. The trend underlines the complex interplay of various factors influencing free-flow capacity over time.

Simultaneously, the queue emission rates exhibited considerable fluctuation, primarily due to the high-resolution congestion detection performed every minute. This granular approach to data collection provides a detailed and dynamic insight into the congestion trends. From 2013 onwards, the queue emission rate manifested an increasing trend until it peaked at roughly 2200 vehicles per hour per lane. Following this peak, the rate remained largely constant, showing negligible variation. This plateauing trend could be attributed to the development of traffic management strategies or the adoption of smart transport systems over the years.

In general, our findings point towards a decreasing trend in free-flow capacity over time, implying a decrease in the efficiency of road utilization. On the contrary, the queue emission rates showcase an initial increase, followed by a largely unchanged pattern. This contrast highlights the importance of long-term monitoring and adaptive management in traffic systems to optimize road usage, reduce congestion, and ensure efficient transport networks. These insights pave the way for future research focusing on exploring the root causes of these trends and developing effective strategies to enhance road network performance.

### 3.3 Analysis of the change in road capacity

This section builds on the previous study by expanding the research sample to analyse the factors that affect road capacity. The RBF algorithm was used to analyse data from 100 roads to find the effect of each factor on the change in road capacity over time.

#### 3.3.1 Data source

In this study, road traffic data for 100 different locations in China during the period 2015–2019 were obtained, all of which are publicly available, and the previous algorithm needed to be modified as the vehicle directions could not be determined due to the constraints of the observations. The data contains basic information about the road section including 12 parameters, as well as data on vehicle movements during the time period, including speed and time of passage.

#### 3.3.2 Introduction to the algorithm

*3*.*3*.*2*.*1 Data pre-processing*. In order to improve the efficiency of the analysis, the data was examined before using the RBF algorithm to eliminate abnormal observations, such as those with a vehicle length of 0 or greater than 20 meters.


$$s^{*}(v)=s_{0}+Tv$$ (3)

v = velocity, T = time interval, s0 = vehicle length and s*(v) is the baseline data. When selecting data using Eq (2), anomalous observations are filtered out based on the relationship between speed and distance. If there are very small data s < s*(v), s < s*(v), when S0 = 0 m and T = 1 s. If there are extremely large data s>s*(v), when S0 = 19m (our lorry length limit is 18m) and T = 4.5s, both do not fit the reality and can be regarded as abnormal data for rejection. At the same time, in order to improve the efficiency of the operation, the time period of high speed driving can be excluded, so for each section of road set a threshold value of 70% of the speed limit, it is considered that no congestion occurs when 70% of the speed limit and above is reached, and then the road capacity of this data set is derived. The statistically relevant parameters are listed in Table 1.

**Table 1:** Parameter properties at a glance.

Serial number	Abbreviations	Coefficient (unit)
1	Time	Hour
2	C.D	Presence of blockage detection and messages
3	Lane	Number of lanes
4	D_export_	Distance from ramp (m)
5	D_ramp_	Distance from ramp (m)
6	P_truck_	Proportion of lorries
7	P_traffic jam_	Congestion Ratio
8	AADT	Annual average daily traffic volume
9	VMB	Availability of variable information panels
10	V_lim_/V_slim_	Presence of speed limits and zone limits
11	T.L	Availability of lanes for lorries
12	Tunnel/Bridge	Tunnel or Bridge

The statistical parameters obtained from the analysis are presented in Table 2.

**Table 2:** List of parameter weight values.

	Numerical values	*c*_1_	*c*_3_	*c*_6_	*c*_7_	*c*_8_	*c*_9_	*c*_10_	*c*_11_
		Time	Lane	P_truck_	P_traffic jam_	AADT	VMB	V_lim_/V_slim_	T.L
Weight (%)	1973	3	-5	-8.1	6.5	3.2	-2.2	-3.3	3.6

Note: For definitions of acronyms, please see Table 1

*3*.*3*.*2*.*2 RBF algorithm*. Radial Basis Function (RBF) neural networks are simple, fast converging networks that can approximate arbitrary non-linear functions. Compared to the global approximation of BP (Back Propagation) neural networks, RBF neural networks are local approximation networks, which are very suitable for applications where real-time performance is important. Common radial basis functions are the Gauss distribution function, the Multi-Quadric inverse function and the thin-plate spline function, all of which are radially symmetric and have a rapid drop in function value after an off-centre position. The RBF algorithm constructs the regularization network firstly and then trains the algorithm.

1. **Regularization Network**
  Regularized networks are typical of radial basis neural networks and generally consist of three layers, namely the input layer, the hidden layer and the output layer, as shown in Figs 4–2, There are M input nodes in the network, Dimension is m; The number of implied layers is the same as the number of training data points. The basis function for the i-th node is $\phi (\left\|X-X_{i}\right\|),X_{i}=[x_{i1},x_{i2},\cdots ,x_{im}]$ is the centre of the basis function, *W*_*ij*_ is the weight value; The output layer has J neurons. Let K = N training samples, Output $Y_{k}=[y_{k1},y_{k2},y_{k3},\cdots ,x_{kJ}]$, When inputting training samples *X*_*k*_,The j-th output results in:
$$y_{kj}=\sum_{i}w_{ij}•\phi (X_{k},X_{i}),i=1,2,\cdots ,Nj=1,2,\cdots ,J$$ (4)
  Using the Gaussian function as the basis function in general, it follows that
$$\phi \left(X_{k},X_{i}\right)=G\left(X_{k},X_{i}\right)=\exp(-\frac{1}{2\sigma^{2}}\|X_{k}-X_{i}\|)$$ (5)
  The number of neural units in the hidden layer can be reduced (that is eliminating factors with less influence) by the Galerkin method when performing analysis of road capacity influencing factors, effectively improving computational efficiency, called generalized radial basis networks, whose model is shown in Eq 6.
$$y_{kj}=w_{0j}+\sum_{1}^{I}w_{ij}\phi \left(X_{k},X_{i}\right),j=1,2,\cdots ,J$$ (6)
  *w*_0*j*_ corresponding to an implicit layer threshold of 0 for the number of nodes in the implicit layer, *I*<*K*.
2. **Algorithm training**
  According to the principle of RBF neural network, the essence of algorithm learning is to find the centre of the basis function associated with the implicit layer, the standard deviation of the basis function and the weight between the implicit layer and the output layer, here refers to the influence weight of each influencing factor on the road capacity. Common algorithms include the self-organized centre selection method, the random centre selection method etc. The random centre selection method is used in this paper, and after selecting a set of centres randomly, the standard deviation can be obtained as follows.
$$\sigma =\frac{d_{\max}}{\sqrt{2n}}$$ (7)
  *d*_max_ denotes the maximum distance from the selected centre, *n* denotes the number of implied nodes. After determining the centre and standard deviation, the basis function (in the case of a Gaussian function) can be obtained as follows:
$$\phi (\left\|X_{k},X_{i}\right\|)=\exp(-\frac{1}{2\sigma^{2}}\|X_{k}-X_{i}\|)=\exp(-\frac{n}{{d_{\max}}^{2}}{\left\|X_{k}-X_{i}\right\|}^{2})$$ (8)

From Eqs (4–6), it is clear that to obtain the final output, need to derive the weight w, Let *d* = {*d*_*kj*_} be the desired output, denotes the expected output value of the k-th input vector at the j-th output node, then the output weight matrix can be derived from the following equation. The pseudo-inverse *G*^+^ can be found by singular value decomposition (SVD), the influence weights of each factor can then be found.

Based on the above analysis, the radial basis neural network showed relatively good performance in dealing with non-linear fuzzy recognition. In terms of input layer data, a matrix is created to collect data in combination with road section attributes, including time, presence of blockage detection and information, number of lanes distance from ramp (m), distance from ramp (m), proportion of trucks, proportion of congestion, annual average daily traffic volume, presence of variable intelligence panels, presence of speed limit and interval speed limit, presence of lane for trucks, presence of bridge and tunnel, and output layer is road capacity. The training sample values required can be obtained by observing traffic volumes and vehicle speeds. In the calculation of road capacity parameters based on radial basis neural networks, the input data should be used partially as training samples.Finding the most suitable number of nodes in the hidden layer by convergence tests of the output results, followed by calibration of the parameter weights of the remaining sections by the trained radial basis neural network. Convergence is judged by comparing the output of the test sample with the actual value, and is generally considered to have converged within 5% error. In order to simplify the computational analysis, parameters with weight coefficients less than 2% in absolute value were excluded, parameters that have a high impact on road capacity were retained.

### 3.4 Parameter analysis

The results of the RBF analysis are shown in Table 2. The road capacity base data is 1973 vehicles per hour per lane, which is generally consistent with our highway design manual. Analysis of the impact of important parameters and their symbols: more lanes will reduce the capacity of an individual road after congestion, because there are more lane changes when there are more lanes, which reduces the efficiency in the use of roads. A higher proportion of lorries will reduce the road capacity after congestion, in line with the general perception that lorries start slower, generally between 15% and 30% of lorries are common on our highways, and this proportion will be increased at night. As the congestion ratio increases, there will be some increase in road capacity, as drivers will become more accustomed to working in such conditions, with shorter vehicle-to-car spacing and vehicle start-up times. An increase in annual average daily traffic (AADT) means more vehicles on the road, and as drivers become accustomed to the busy environment, they can travel at higher speeds with less impact; Road warning message signs such as variable message boards reduce road capacity slightly as drivers need to observe and thus reduce their speed slightly, and if they make lane changes as a result of the warning message they also reduce road capacity. Both speed measuring devices reduce road capacity slightly. Drivers tend to habitually reduce their speed to pass when they see a speed measuring device. Dedicated lanes for lorries will increase road capacity, but the lorry lane capacity will be reduced. Observing the same road on an annual scale, the capacity has increased every year. However, the magnitude is smaller. The specific numerical relationships are required further analysis and cannot be explained by the variables in the table alone.

## 4 Conclusion

This study provides a comprehensive analysis of changes in free-flow capacity and queue discharge rates over a ten-year period for road sections where basic conditions remain consistent. Further, it employs the Radial Basis Function (RBF) algorithm to study data relating to 100 roads to dissect the impact of various factors on road capacity over time.

1. Utilizing the K-M method for roads with constant base conditions, this study explores the road movements over a decade. Our findings underline a gradual reduction in capacity over time on the same road, mirroring the perceived capacity decrease as the road’s condition deteriorates due to extended usage. For example, we noticed a reduction from an average capacity of 2000 vehicles per hour per lane in 2013 to approximately 1800 vehicles per hour per lane in 2023, representing a 10% decrease over a decade. These results offer vital insights for traffic management and road planning authorities. Upon the onset of congestion, a small increase in road capacity was observed, and then it remained largely unchanged. This shows that while initial response to congestion might enhance the throughput, the system tends to reach a saturation point and remains relatively stable thereafter.
2. Using the RBF algorithm, the influence of various factors on road capacity was analyzed. By examining data from 100 road sections, 12 parameters potentially affecting road capacity and their reasons were scrutinized. Key observations include: An increase in the number of lanes led to a reduction in the overall average lane capacity of the road, possibly due to the lane-changing behaviors. An increase in the proportion of lorries correlated with a reduction in road capacity, likely due to their lower speeds and larger sizes. Interestingly, an increase in the proportion of congestion resulted in a slight increase in road capacity, perhaps due to improved driver awareness and more efficient use of the road during these periods. The installation of various cues and speed measuring devices generally led to a decrease in road capacity, indicating that the control measures could potentially disrupt the traffic flow. Dedicated lanes for lorries had a positive impact on road capacity, hinting at the effectiveness of segmentation in improving road utilization.
3. Although this study provides valuable insights, it is primarily based on limited data. Further validation and refinement can be undertaken with larger road data sets, providing more robust and reliable conclusions. Additionally, given that variations in road capacity are highly influenced by driver attributes and vehicle performance, future research in these areas could offer more nuanced understandings of the intricate dynamics governing road traffic capacity. This would significantly contribute to the development of more effective and targeted traffic management and planning strategies.

## Supporting information

S1 Data Traffic flow and traffic jam probability distribution function. (XLSX)

S2 Data Distribution of traffic at different times. (XLSX)

S3 Data Variation in road free flow capacity. (XLSX)

S4 Data Changes in road queue emission rates. (XLSX)
